# Recombinant Viral Vaccines Expressing Merozoite Surface Protein-1 Induce Antibody- and T Cell-Mediated Multistage Protection against Malaria

**DOI:** 10.1016/j.chom.2011.03.002

**Published:** 2011-03-17

**Authors:** Simon J. Draper, Anna L. Goodman, Sumi Biswas, Emily K. Forbes, Anne C. Moore, Sarah C. Gilbert, Adrian V.S. Hill

**Affiliations:** 1 The Jenner Institute, University of Oxford, Old Road Campus Research Building, Oxford OX3 7DQ, UK

The study was supported by funding from the NIHR Oxford Biomedical Research Centre program.

